# Supplementary material for: Fear extinction is regulated by the activity of long noncoding RNAs at the synapse
Source: Nat Commun. 2023 Nov 22;14:7616. doi: 10.1038/s41467-023-43535-1 (PMC10665438; doi:10.1038/s41467-023-43535-1)
Supplement: Supplementary file 1 — Supplementary Information [file 41467_2023_43535_MOESM1_ESM.pdf]

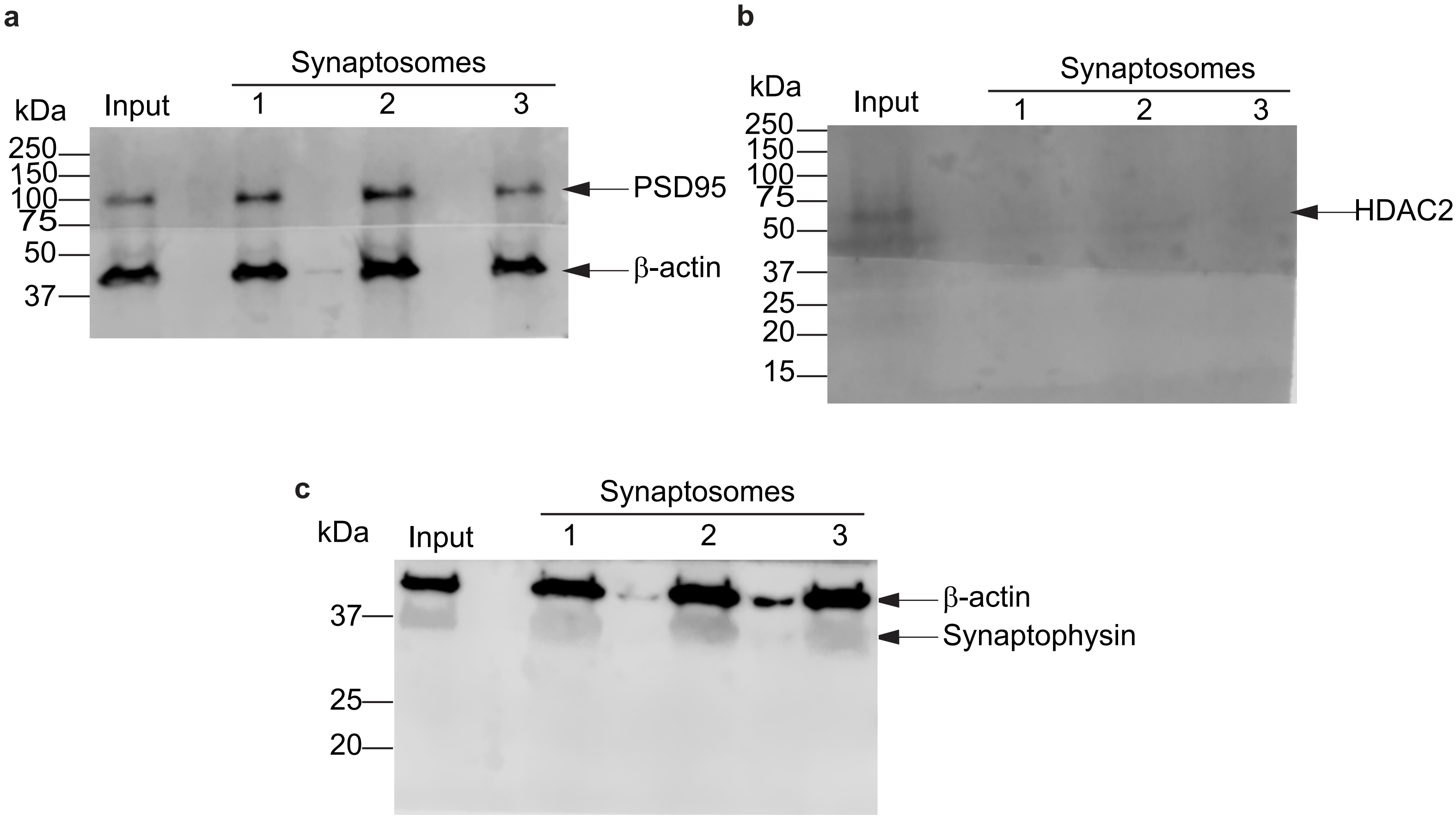

Supplementary Figure 1. Blot displaying a) PSD95 b) HDAC2 and c) synaptophysin proteins from synaptosomes of mouse prefrontal cortex (PFC). Each lane represents separate synaptosome protein lysates of four PFCs. β-actin is used as the internal loading control. 50 µg of protein lysates were loaded onto each lane. The molecular marker and 10% input are indicated.

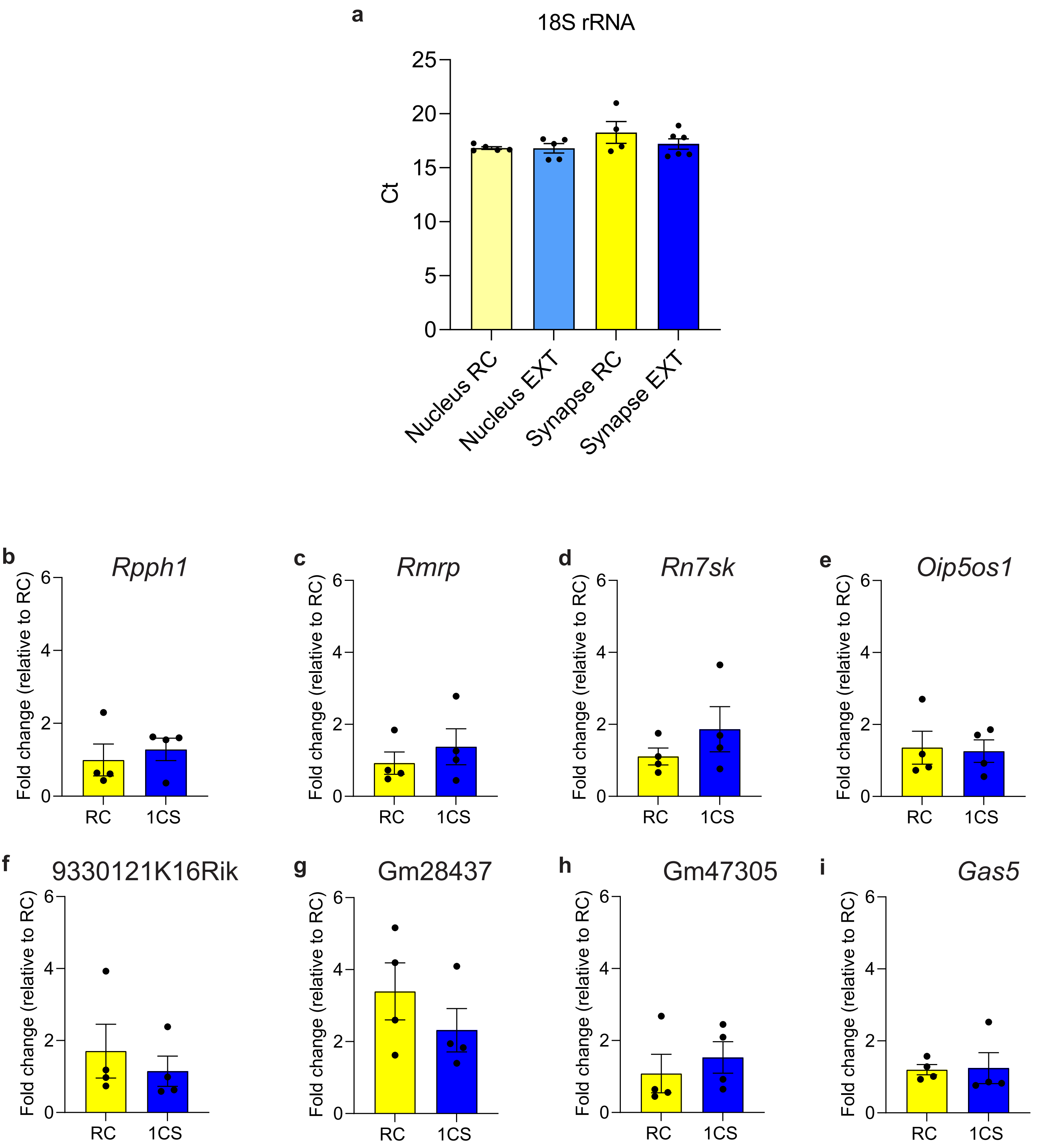

Supplementary Figure 2. (a) Graph showing RT-qPCR Ct values of housekeeping gene 18S rRNA in the nucleus and synapse in the ILPFC following 60CS fear extinction training (EXT) (nucleus EXT, n = 5 independent biological replicates per group; synapse EXT, n = 6 independent biological replicates per group). Retention control (RC) is also indicated (nucleus RC, n = 5 independent biological replicates per group; synapse RC, n = 4 independent biological replicates per group). Statistical significance was determined using a two-tailed unpaired Student's t-test.(b-i) RT-qPCR of 8 of the 10 candidates in the synapse in the ILPFC following a short 1CS fear extinction training. 18S rRNA was used as the housekeeping gene for normalization. n = 4 biological replicates per group. Statistical significance was determined using a two-tailed unpaired Student's t-test.

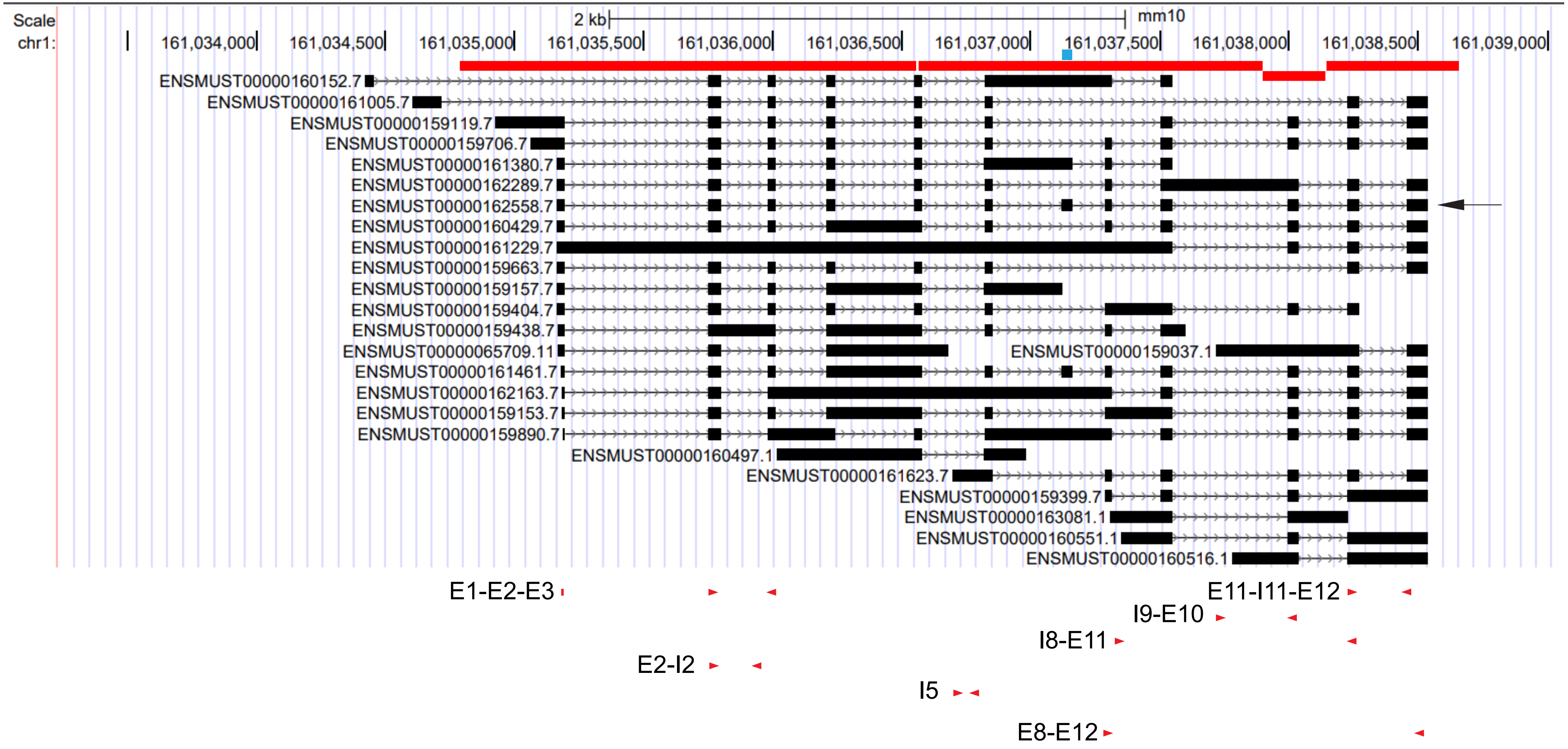

Supplementary Figure 3. Genomic track displaying *Gas5* locus. All 25 *Gas5* variants are plotted below the track. Arrow indicates *Gas5* variant ENSMUST00000162558.7. Bars shown in red and blue are *Gas5* capture-seq probes and gRNA target region, respectively. Arrow heads indicate primers used in Figure 2e.

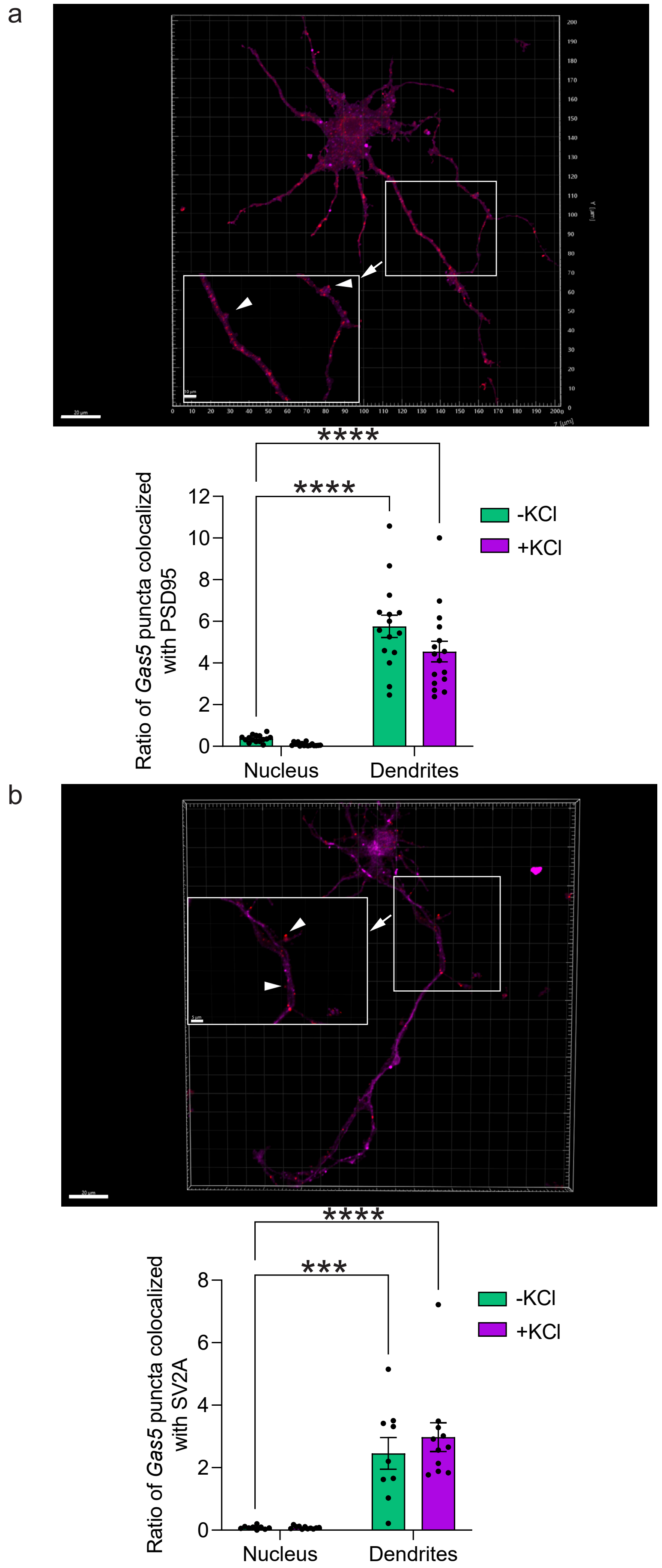

Supplementary Figure 4. Representative image showing the co-loocalized expression of the *Gas5* variant with the synaptic marker a) PSD95 and b) SV2A in primary cortical neurons. Arrowheads show co-localized *Gas5* expression at the dendritic spine. Scale bar, 20 µm. Red represents *Gas5*; magenta represents a) PSD95 or b) SV2A protein. The boxed region is enlarged in the inserts. Scale bar, 10 µm (PSD95), 5 µm (SV2A). Graph showing ratio of *Gas5* puncta colocalized with a) PSD95 (-KCl, n = 15 neurons, +KCl, n = 16 neurons, two-way ANOVA, $F_{1,58}$ = 182.1, p<0.0001; Dunnett's post hoc tests: nucleus -KCl versus dendrites -KCl, ****p<0.0001, nucleus -KCl versus dendrites +KCl, ****p<0.0001) or b) SV2A (-KCl, n = 9 neurons, +KCl, n = 11 neurons, two-way ANOVA, $F_{1,36}$ = 59.31, p<0.0001; Dunnett's post hoc tests: nucleus -KCl versus dendrites -KCl, ***p<0.001, nucleus -KCl versus dendrites +KCl, ****p<0.0001) versus total number of non-colocalized puncta. Error bars represent S.E.M.

Supplementary Figure 4

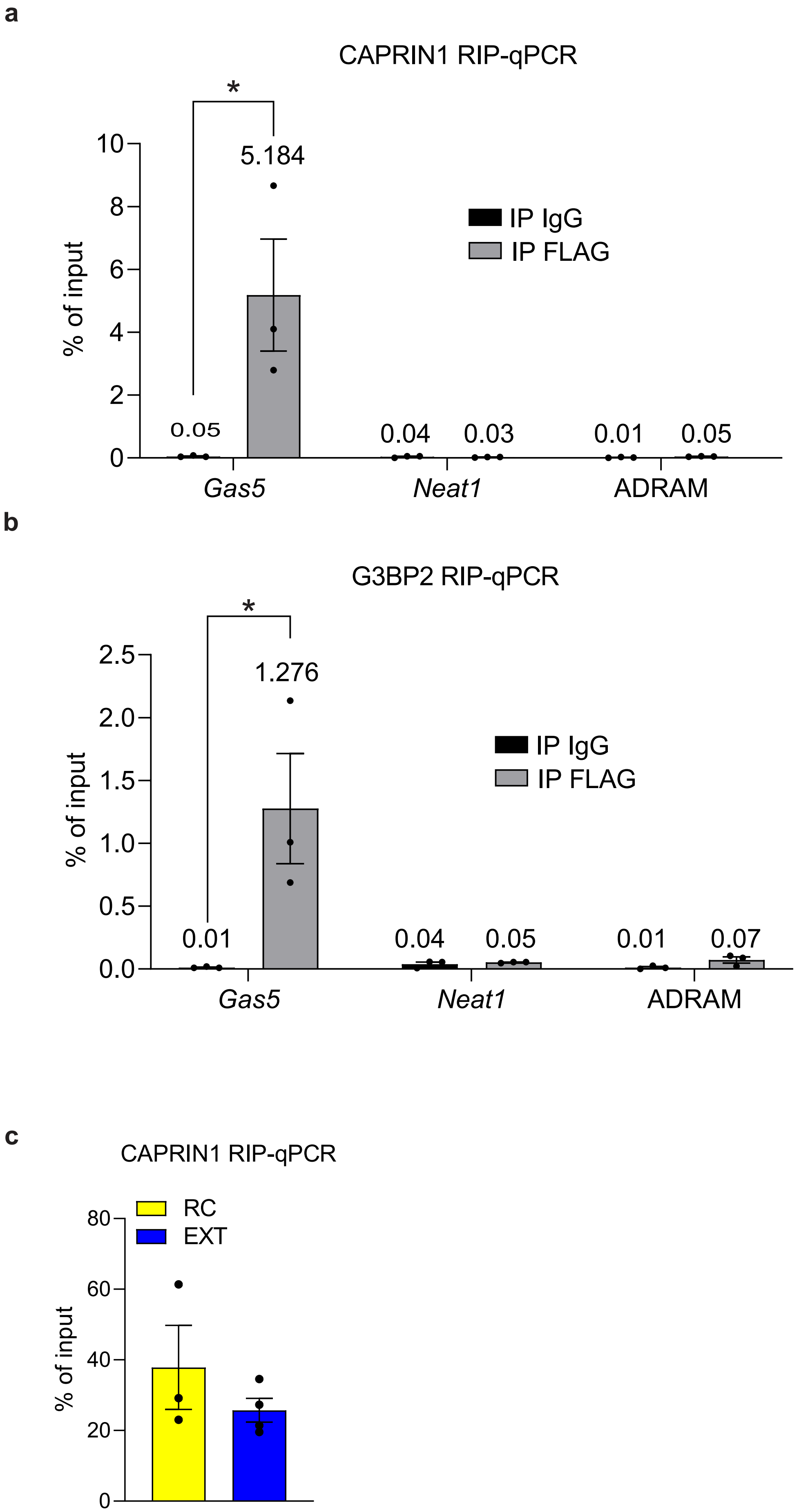

Supplementary Figure 5. *Gas5* RIP-qPCR of primary cortical neurons expressing FLAG-tagged a) CAPRIN1 or b) G3BP2. Percent of input is shown. Rabbit IgG control is used as control. Expression of lncRNA controls, ADRAM and *Neat1*, are indicated. n = 3 biological independent replicates. Statistical significance was determined using two-tailed unpaired Student's t-test (CAPRIN1, $t(4) = 2.884$, $p = 0.0448$; G3BP2, $t(4) = 2.884$, $p = 0.0448$). *$p<.05$. Error bars represent S.E.M. c) *Gas5* CAPRIN1 RIP-qPCR of mice following fear extinction training (EXT) (n = 4 biological independent replicates per group). Retention control (RC) (n = 3 biological independent replicates per group) and percentage of input are indicated. Statistical significance was determined using two-tailed unpaired Student's t-test. Error bars represent S.E.M.

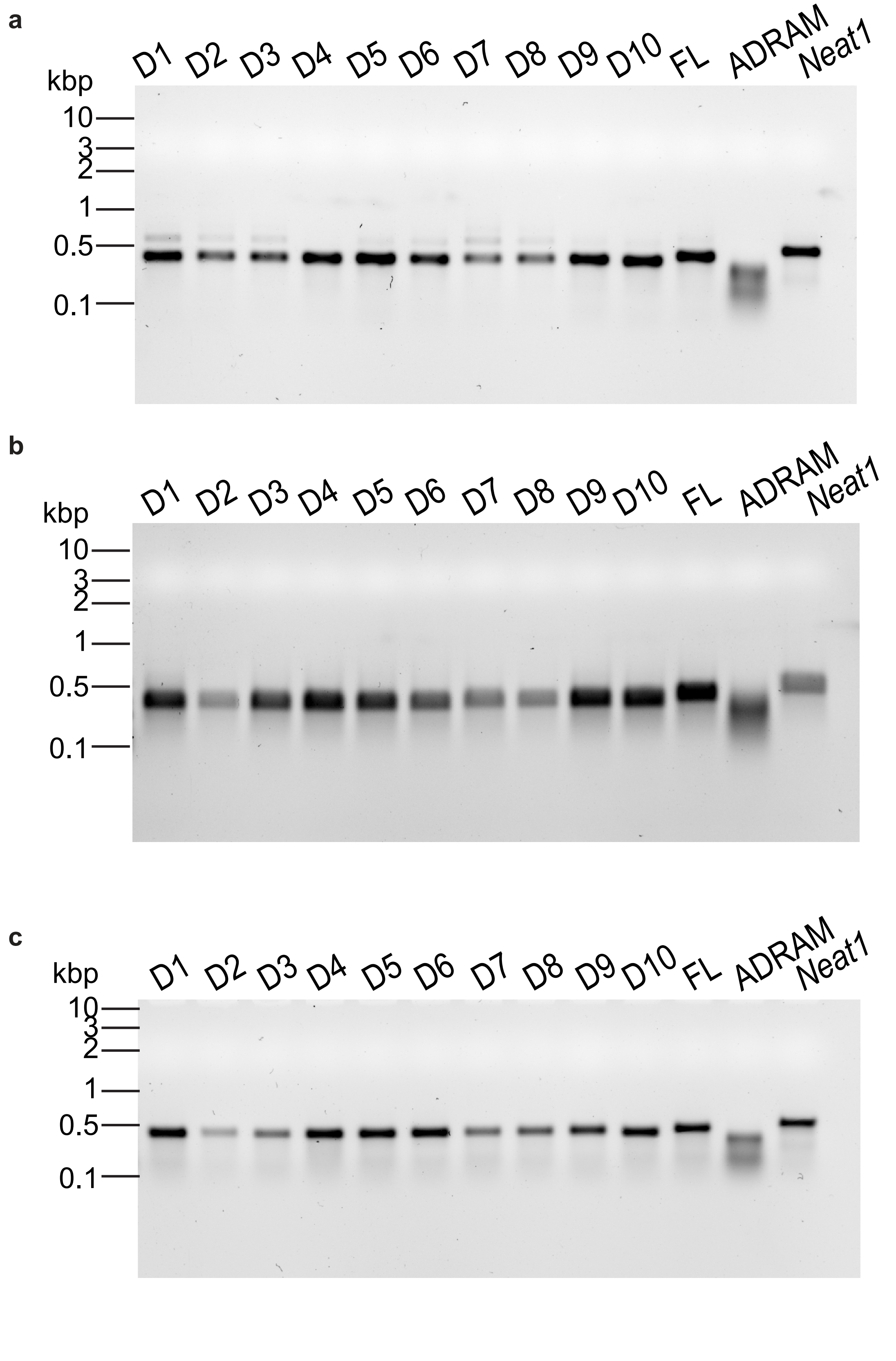

Supplementary Figure 6. RNA native gel (1%) displaying 1 μg of mutant (D1-D10) and full-length (FL) *Gas5* RNAs a) before and b) after in-vitro RNA folding assay. Negative control RNAs, ADRAM and *Neat1*, are indicated. c) RNA native gel (1%) showing mutant (D1-D10) and full-length (FL) *Gas5* RNAs isolated after incubating with ILPFC protein extracts for 2 hr. Negative control lncRNAs, ADRAM and *Neat1*, are indicated.

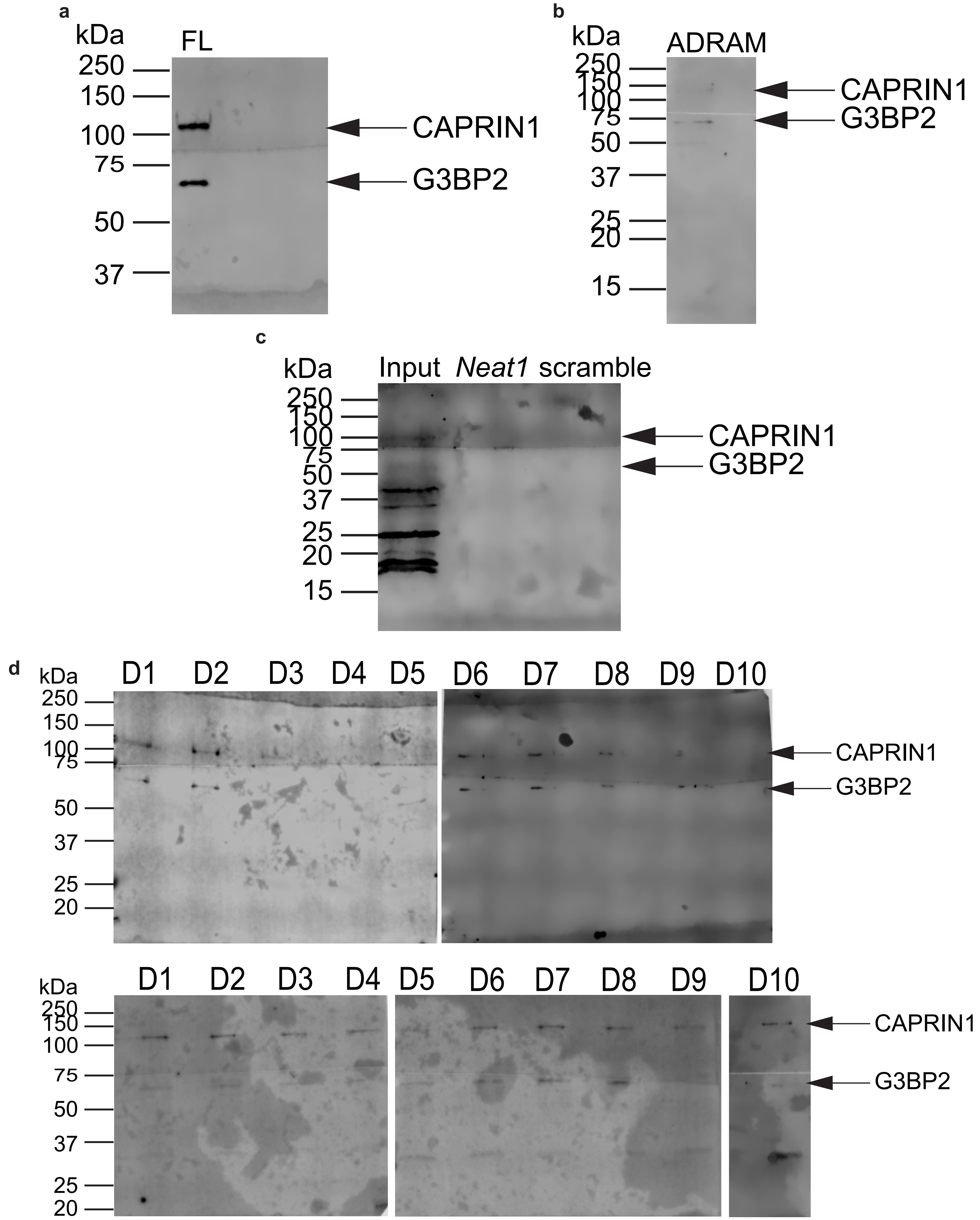

Supplementary Figure 7. Blots displaying CAPRIN1 and G3BP2 proteins after incubating a) full-length in-vitro transcribed *Gas5* or b) ADRAM or c) *Neat1* with ILPFC protein extracts. Input and scramble RNA control (5 -CCUGGUUUUUAAGGAGUGUCGCCAGAGUGCCGCGAAUGAAAAA-3) are indicated. d) Blots displaying CAPRIN1 and G3BP2 proteins after incubating different fragments of in vitro transcribed *Gas5* with ILPFC protein extracts. Band intensity was quantified and plotted in Figure 4c and 4d.

Supplementary Figure 7

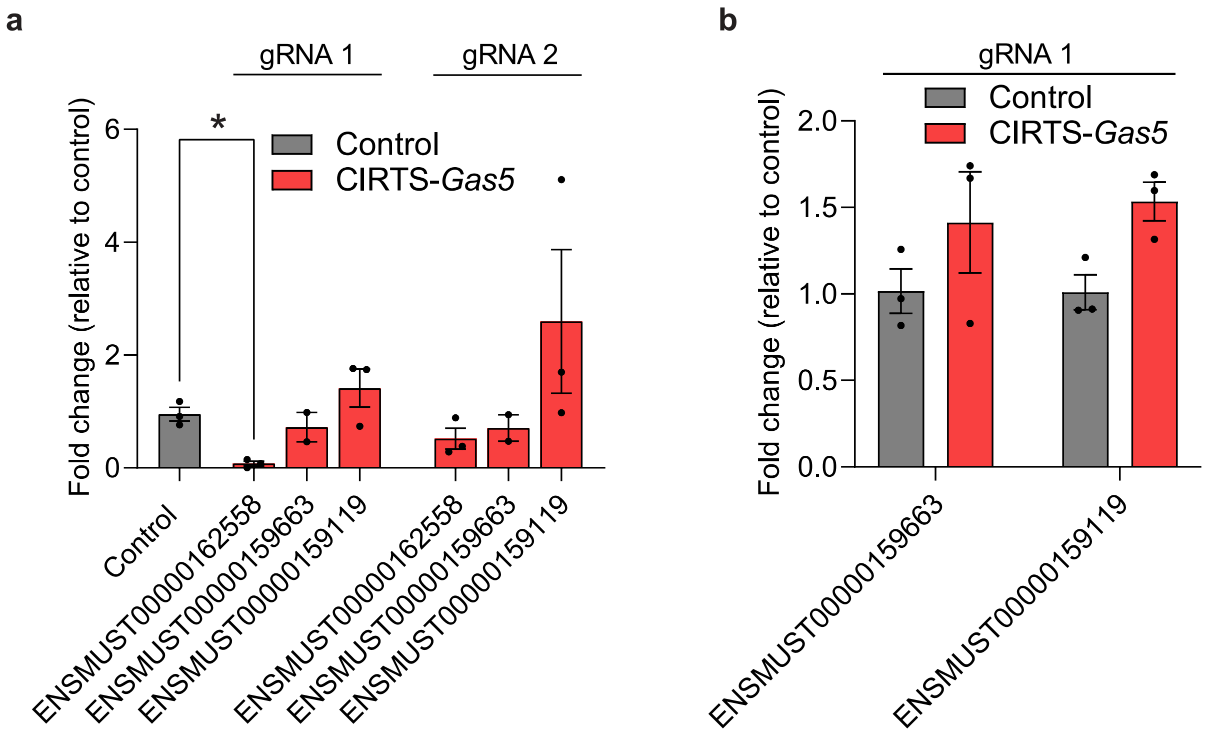

Supplementary Figure 8. a) In-vitro nuclease cleavage assay with two guides targeting *Gas5* variant ENSMUST00000162558. Guide 1 was used in the experiments described in Figure 5 and 6. *Gas5* knockdown was assessed by RT-qPCR. *Gas5* splice variants are indicated. n = 3 independent biological replicates per group. Statistical significance was determined using a two-tailed unpaired Student's t-test $t(2.497) = 6.837$, $p = 0.0112$. *$p<.05$. Error bars represent S.E.M. b) To verify the specificity of the CIRTS-*Gas5* knockdown construct, qRT-PCR was performed on primary cortical neurons transduced with either control or CIRTS-*Gas5* virus revealing no effect on non-target *Gas5* splice variants ENSMUST00000159663 and ENSMUST00000159119. n = 3 independent biological replicates per group. Error bars represent S.E.M.

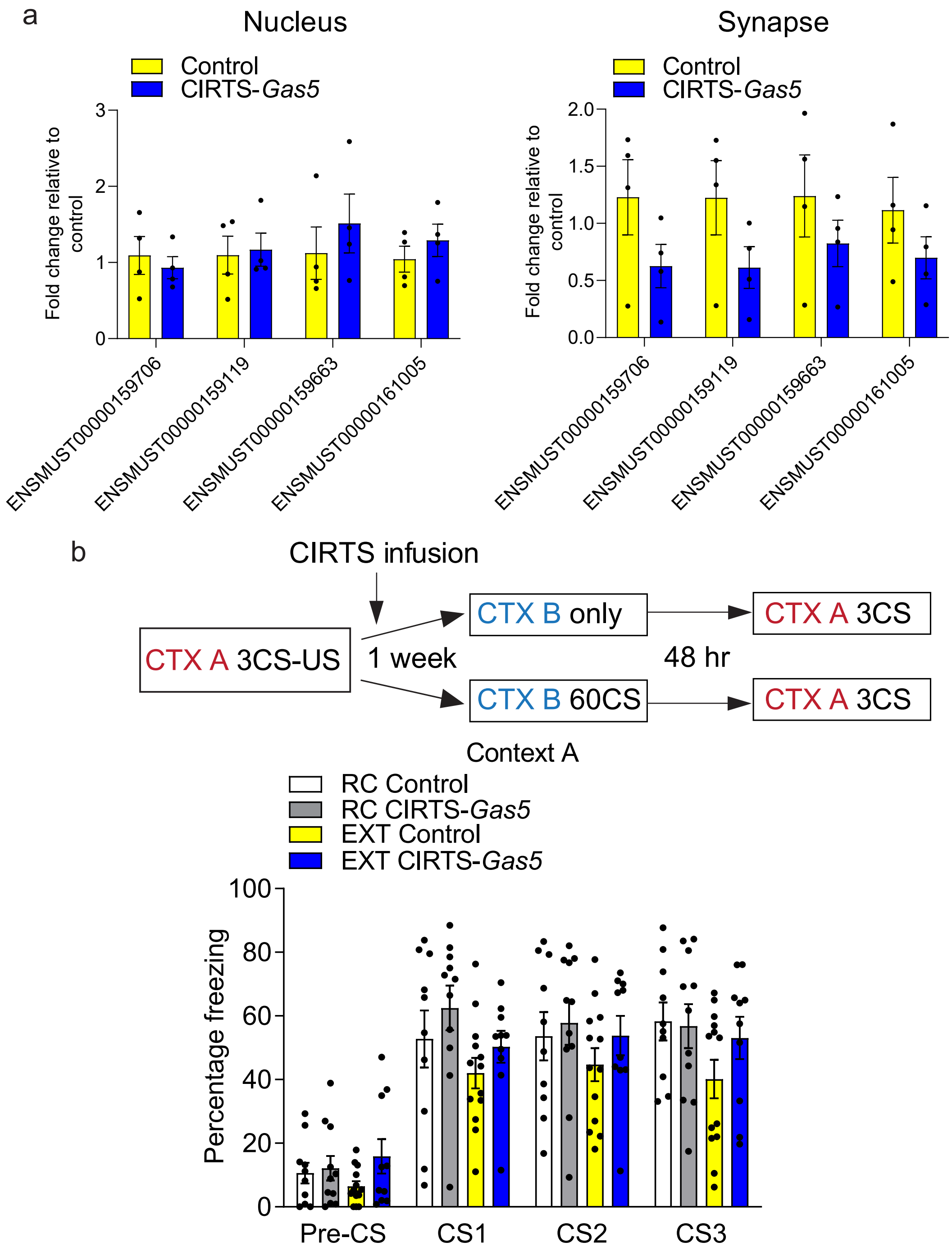

Supplementary Figure 9. a) RT-qPCR revealed no effect of targeted *Gas5* knockdown on other *Gas5* splice variants in the nucleus and at the synapse in the ILPFC. n = 4 independent biological replicates per group. Statistical significance was determined using a two-tailed unpaired Student's t-test. all p>0.05. Error bars represent S.E.M. b) There was no significant effect of *Gas5* knockdown on fear memory when tested in Context A (RC control, n = 10 independent biological replicates per group, RC CIRTS-*Gas5*, n = 11 independent biological replicates per group, EXT control, n = 13 independent biological replicates per group, EXT CIRTS-*Gas5*, n = 8 independent biological replicates per group, two-way ANOVA, $F_{3,38}$ = 1.563, p = 0.2143; Dunnett's post hoc tests: all p>0.05). Error bars represent S.E.M.

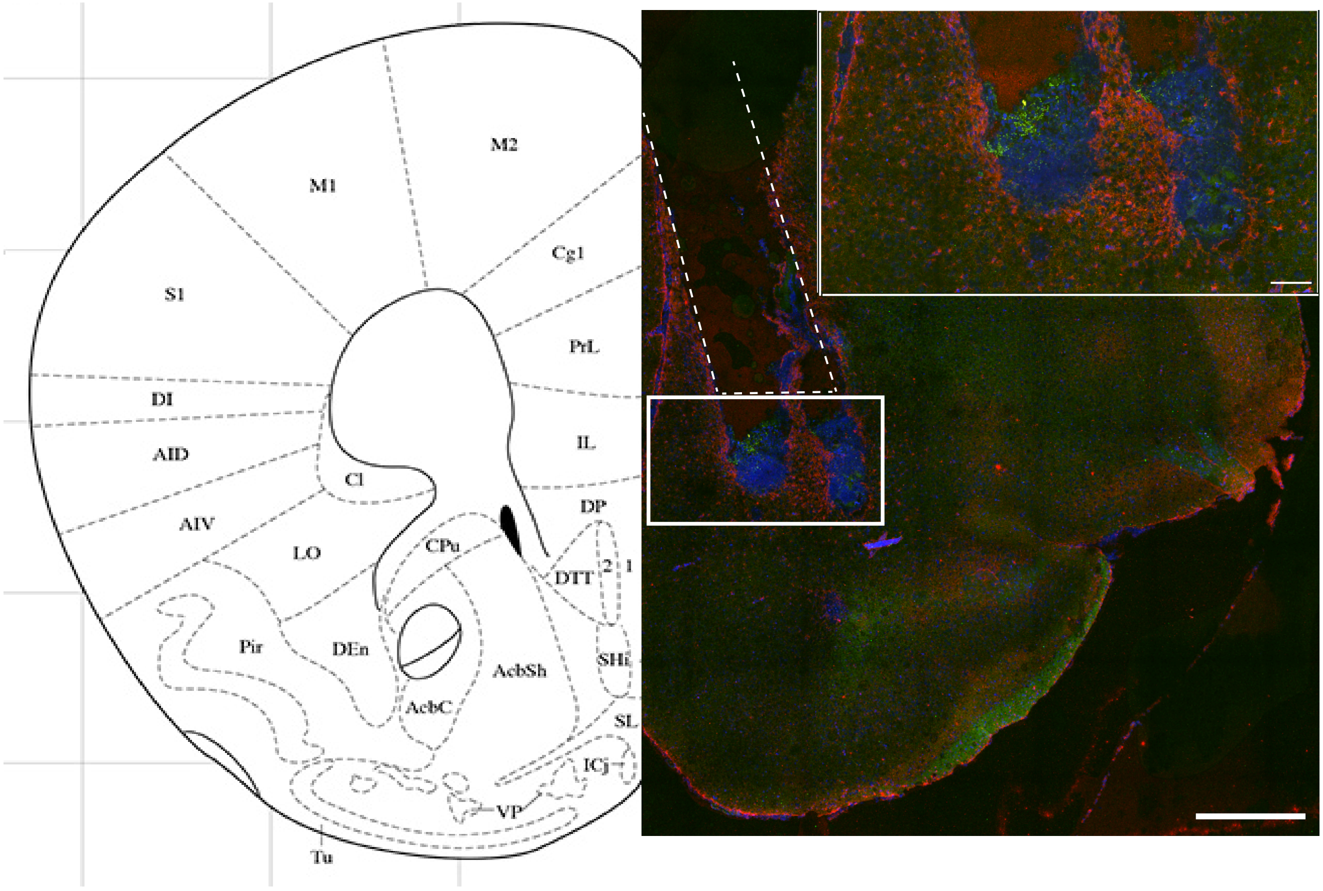

Supplementary Figure 10. Left: representative image of cannula placement in the ILPFC, right: transduction of CIRTS-*Gas5* into the ILPFC of extinction-trained animal. Dotted lines represent the cannula track. Scale bar, 500 µm. Red, *Gas5*; blue, DAPI; green, CIRTS. The boxed region is enlarged in the inserts. Scale bar, 100 µm.

a

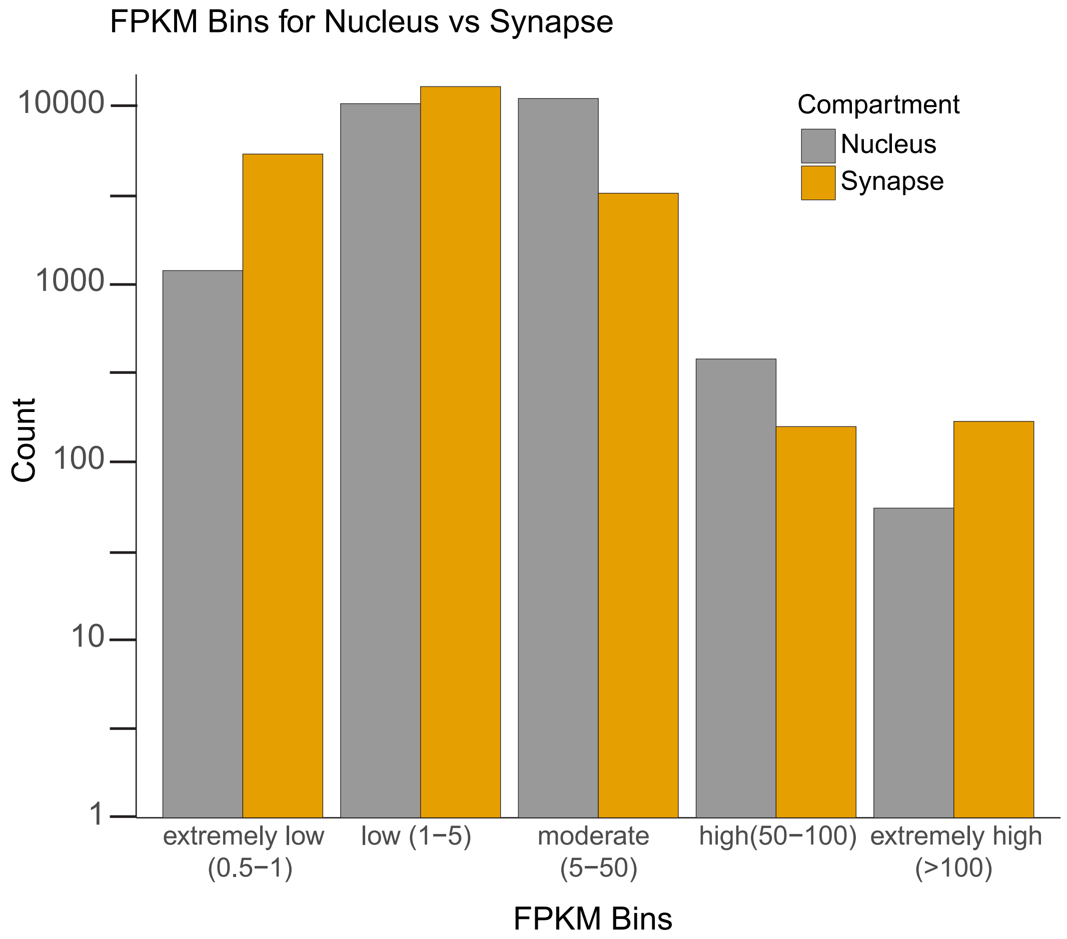

b

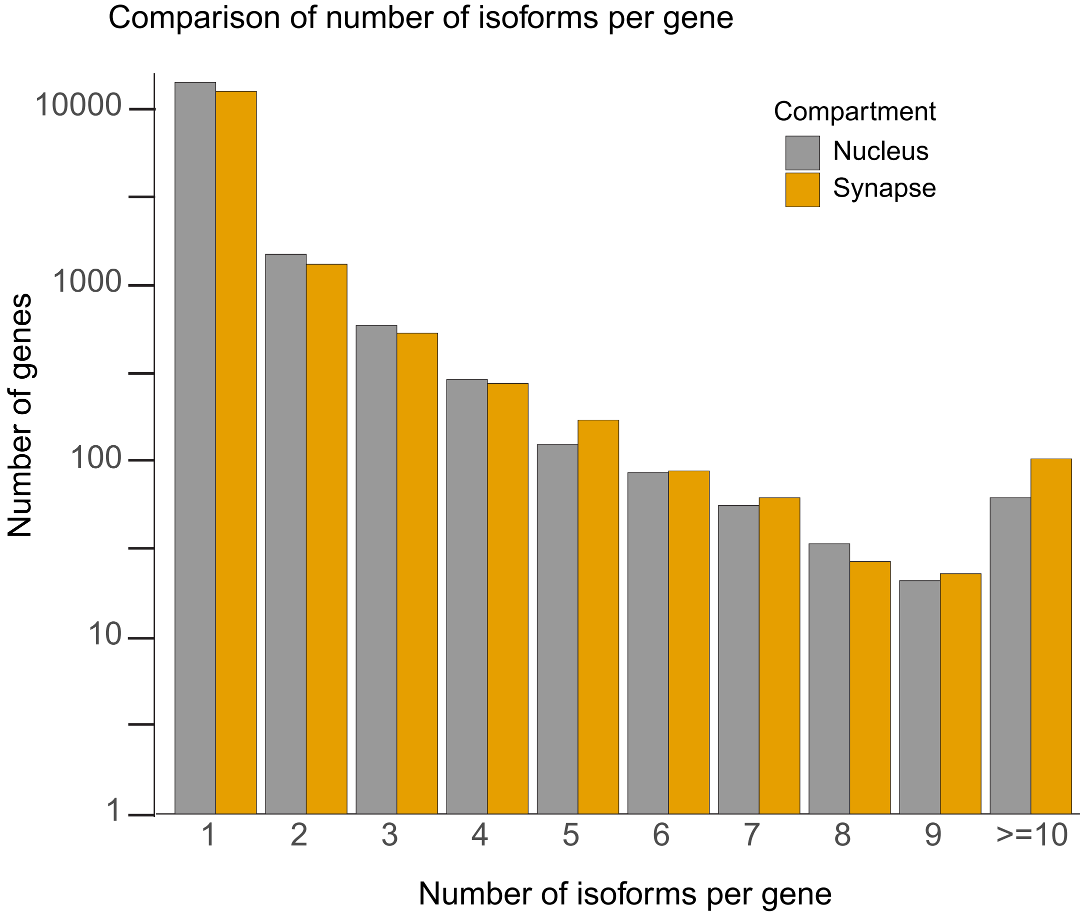

Supplementary Figure 11. a) Distribution of FPKM counts from nucleus and synapse capture-seq data in five categories: i) extremely low expression (0.5 < FPKM <= 1), ii) low expressed (1 < FPKM <= 5), iii) moderate expression (5 < FPKM <= 50), iv) high expression (50 < FPKM <= 100), and v) extremely high expression (FPKM > 100). b) Distribution of the number of isoforms per gene for transcripts with an FPKM > 0.5 in nucleus and synapse capture-seq data.

Supplemental Table 1

Primers and CIRTS gRNA sequence

Primers for quantitative PCR

Name	Forward Sequence (5'-3')	Reverse Sequence (5'-3')
Exon 11 - Intron 11 - Exon 12	GCTCCTGTGACAAGTGGACA	GCACCTGCAACAACCAACAG
Intron 9 - Exon 10	CTGTGCCACTGACTTAACCC	TCAGAATGAAGGACCGGAAA
Intron 8 - Exon11	AGGTACTGTTAGTGATGATC	ATGTCCACTTGTCACAGGAG
Exon 2 - Intron 2	AATGGCAGTGTGGACCTCTG	TCAGTCCCCTTCTTCACGGA
Intron 5	GAACAAATACTGACTACCTG	TGCCCCACAATAAATGTCAA
Exon 1 - Exon 2 - Exon 3	ATTCTGAGCAGGAATGGCAG	TCCTCCTTTGCCACAGAACT
Exon 8 - Exon 12	GTACAAATAATGGTTTGAAT	TGTTATAATACACTTTAATG
Gas5	GGAAGCTGGATAACAGAGCGA	GCATGCAACCAGTTAACTTTCA
18S rRNA	CTGGATACCGCAGCTAGGAA	GAATTTCACCTCTAGCGGCG
Pgk	TGCACGCTTCAAAAGCGCACG	AAGTCCACCCTCATCACGACCC
Rpph1	CTCTACGCTTGGGCAGAC	CTCACCTCAGCCATTGAACT
Rmrp	CCTGTTTCCTAGGCTACATACG	GCGGGCTAACAGTGACTT
Rn7sk	TCGGTCAAGGGTATACGAGTAG	TTTGGATGTGTCTGGAGTCTTG
9330121K16Rik	AGTAGAGTTAGGGTGGGATAGG	ACAGGCACTGATGTGAGTTAG
Gm28437	CAGGATTCTTCTGAGCGTTCTAT	TGGGACTTCTAGAGGGTTAAGT
Gm47305	AGACAGGAGGATCGCTTGA	TCACCATATTGATGCCGAACTTA
Cyrano	GGCTCCATAGAAGCGACATAC	CCCAAGAGCTGGGCATAAA
Meg3	ATTAGGCCAAAGCCATCATCT	GGCGCTTCCAATCGATTTAC
ENSMUST00000159663.7	GGAAGCTGGATAACAGAGCGA	CACAGGAGCCCTTTCAAACT
ENSMUST00000159119.7	TGAAAGGTATTAATGGGTCACCTC	TCTGACACCATCTTCTATTTGAGC
ENSMUST00000161005.7	GAAGGTCGCCGAGTGCT	GAGATCCCACAAGATGTCCCAT

Primers for *in-vitro* Transcription

Name	Sequence (5'-3')
T7 *Gas5* del1-50 Forward	TAATACGACTCACTATAGGGTGATGGGACATCTTGTG
T7 *Gas5* Forward	TAATACGACTCACTATAGGAGCCTTTCGGAGCTGTGC
Gas5 Reverse	TTCATGTTATAATACACTTT
Gas5 del458-504 Reverse	ATTTGAGCCTCCATCCAGGC
T7 ADRAM Forward	TAATACGACTCACTATAGGTTTGCCTCAACTCCACTA
ADRAM Reverse	GCAGAGGTACAAACTTTC
T7 *Neat1* Forward	TAATACGACTCACTATAGGAAGAAGCTTTAGATGACG
Neat1 Reverse	TTGGCTTGGAAATGTAAG

CIRTS gRNA

Name	Sequence (5'-3')
Gas5 gRNA	AGCAAGCCAGCCAAATGAACAAGCATGCAA
Control Scramble	GATACATCATCTCTGTATTAGGCTCCCAAC
