# Supplementary material for: Fear extinction is regulated by the activity of long noncoding RNAs at the synapse
Source: Nat Commun. 2023 Nov 22;14:7616. doi: 10.1038/s41467-023-43535-1 (PMC10665438; doi:10.1038/s41467-023-43535-1)
Supplement: Supplementary file 3 — Description of Additional Supplementary Files [file 41467_2023_43535_MOESM3_ESM.pdf]

Description of Additional Supplementary Files

File Name: Supplementary Data 1
Description: Transcripts identified in the nucleus and synapse lncRNA capture-seq

File Name: Supplementary Data 2
Description: Synapse-enriched transcripts identified in the lncRNA capture-seq

File Name: Supplementary Data 3
Description: Sheet 1, Transcripts identified in the synapse RC and synapse EXT RNA-seq; Sheet 2, Top10 capture-seq candidates identified in the synapse RC and EXT RNAseq

File Name: Supplementary Data 4
Description: Genomic coordinates for all captured regions in the lncRNA capture-seq

File Name: Supplementary Data 5
Description: Genomic coordinates for the captured Gas5 region in the lncRNA captureseq

File Name: Supplementary Data 6
Description: Sheet 1, Gas5 RIP targets identified in the RC and EXT group; Sheet 2, Gas5 RIP targets identified exclusively in the RC and EXT group, and overlap in both
