# Supplementary material for: Fear extinction is regulated by the activity of long noncoding RNAs at the synapse
Source: Nat Commun. 2023 Nov 22;14:7616. doi: 10.1038/s41467-023-43535-1 (PMC10665438; doi:10.1038/s41467-023-43535-1)
Supplement: Supplementary file 10 — Reporting Summary [file 41467_2023_43535_MOESM10_ESM.pdf]

nature portfolio

Corresponding author(s): Timothy W. Bredy; Wei-Siang Liau

Last updated by author(s): Oct 27, 2023

Reporting Summary

Nature Portfolio wishes to improve the reproducibility of the work that we publish. This form provides structure for consistency and transparency in reporting. For further information on Nature Portfolio policies, see our Editorial Policies and the Editorial Policy Checklist.

Statistics

For all statistical analyses, confirm that the following items are present in the figure legend, table legend, main text, or Methods section.

n/a	Confirmed	
☐	☒	The exact sample size (*n*) for each experimental group/condition, given as a discrete number and unit of measurement
☐	☒	A statement on whether measurements were taken from distinct samples or whether the same sample was measured repeatedly
☐	☒	The statistical test(s) used AND whether they are one- or two-sided<br>*Only common tests should be described solely by name; describe more complex techniques in the Methods section.*
☐	☒	A description of all covariates tested
☐	☒	A description of any assumptions or corrections, such as tests of normality and adjustment for multiple comparisons
☐	☒	A full description of the statistical parameters including central tendency (e.g. means) or other basic estimates (e.g. regression coefficient) AND variation (e.g. standard deviation) or associated estimates of uncertainty (e.g. confidence intervals)
☐	☒	For null hypothesis testing, the test statistic (e.g. F, t, r) with confidence intervals, effect sizes, degrees of freedom and P value noted<br>*Give P values as exact values whenever suitable.*
☒	☐	For Bayesian analysis, information on the choice of priors and Markov chain Monte Carlo settings
☒	☐	For hierarchical and complex designs, identification of the appropriate level for tests and full reporting of outcomes
☒	☐	Estimates of effect sizes (e.g. Cohen's *d*, Pearson's *r*), indicating how they were calculated

Our web collection on statistics for biologists contains articles on many of the points above.

Software and code

Policy information about availability of computer code

Data collection	Animal behavioral data was collected by FreezeFrame version 4 software. IHC and IF images were acquired by ZEN 2012 software and SlideBook version 6.0. Western blot data was collected by Image studio version 5.0 (Licor). Quantitative PCR data was acquired by RotorGene Q software version 2.3.5.
Data analysis	All analysis pipeline are available on GitHub at https://github.com/Qiongyi/lncRNA_nucleus_synapse. Image analysis was performed using the Huygen Professional version 15.05, ImageJ version 1.54f, FigureJ version 1.36 and Imaris version 10.0.1. All statistical analysis was performed by Graphpad Prism version 9. Single molecule data was analyzed by Meta-Morph Microscopy Automation and Image Analysis version 7.7.8, PalmTracer version 2.0.4.1778, SharpVisu version 1.3 and NASTIC version 1. Neurons recordings was analyzed by pClamp version 10.5 and Matlab version R2021b. Softwares used for sequencing data analysis are Cutapt version 1.17, HISAT2 version 2.1.0, SAMtools version 1.8, StringTie version 2.1.4, Ballgown version 2.22.0 and SUPPA2 version 2.3. Isoforms expression was plotted and visualized by IsoVis version 1.1.1 (https://isomix.org). Mass spectrometry data was analyzed by Protein Pilot version 5.0 and Analyst version 1.7.

For manuscripts utilizing custom algorithms or software that are central to the research but not yet described in published literature, software must be made available to editors and reviewers. We strongly encourage code deposition in a community repository (e.g. GitHub). See the Nature Portfolio guidelines for submitting code & software for further information.

Data

Policy information about availability of data

All manuscripts must include a data availability statement. This statement should provide the following information, where applicable:

- Accession codes, unique identifiers, or web links for publicly available datasets
- A description of any restrictions on data availability
- For clinical datasets or third party data, please ensure that the statement adheres to our policy

Protein network analysis was performed using the STRING protein query database version 11.5 (https://string-db.org). All sequencing data are available on GitHub at https://github.com/Qiongyi/lncRNA_nucleus_synapse. The sequencing data generated in this study have been deposited in the NCBI Gene Expression Omnibus database under the accession code GSE207149. Proteomics data generated in this study have been deposited to the ProteomeXchange Consortium via the PRIDE partner repository with the dataset identifier PXD046479. All image data generated in this study are available at Figshare (https://doi.org/10.6084/m9.figshare.24431452). All data generated in this study are provided in the Supplementary information or Source Data file. Source data are provided with this paper (Source Data.xlsx). The raw data that support the findings of this study are also available from the corresponding authors upon reasonable request.

Research involving human participants, their data, or biological material

Policy information about studies with human participants or human data. See also policy information about sex, gender (identity/presentation), and sexual orientation and race, ethnicity and racism.

Reporting on sex and gender	N/A
Reporting on race, ethnicity, or other socially relevant groupings	N/A
Population characteristics	N/A
Recruitment	N/A
Ethics oversight	N/A

Note that full information on the approval of the study protocol must also be provided in the manuscript.

Field-specific reporting

Please select the one below that is the best fit for your research. If you are not sure, read the appropriate sections before making your selection.

☒ Life sciences ☐ Behavioural & social sciences ☐ Ecological, evolutionary & environmental sciences

For a reference copy of the document with all sections, see nature.com/documents/nr-reporting-summary-flat.pdf

Life sciences study design

All studies must disclose on these points even when the disclosure is negative.

Sample size	Sample size was chosen based on those reported in previous publications (PMID: 30778148; PMID: 32367065).
Data exclusions	We excluded animals if they did not reach freezing thresholds of 40% . We also excluded data points from animals that had misplaced cannulas or inefficient viral spread.
Replication	We successfully reproduced all biological replicates.
Randomization	Animals were randomly assigned to groups using Random.org following fear conditioning to ensure each groups has a balanced freezing scores.
Blinding	During behavioral test, data was blinded captured and analyzed by full automatic analysis software. RT-qPCR, Single-molecule imaging, sequencing, Immunofluorescence and behavioral experiments were blindly repeated by the investigators.

Reporting for specific materials, systems and methods

We require information from authors about some types of materials, experimental systems and methods used in many studies. Here, indicate whether each material, system or method listed is relevant to your study. If you are not sure if a list item applies to your research, read the appropriate section before selecting a response.

Materials & experimental systems

n/a	Involved in the study
☐	☒ Antibodies
☐	☒ Eukaryotic cell lines
☒	☐ Palaeontology and archaeology
☐	☒ Animals and other organisms
☒	☐ Clinical data
☒	☐ Dual use research of concern
☒	☐ Plants

Methods

n/a	Involved in the study
☒	☐ ChIP-seq
☒	☐ Flow cytometry
☒	☐ MRI-based neuroimaging

Antibodies

Antibodies used

Primary antibodies used in this study for western blot are as follows: Anti-Caprin1 Antibody (1:2000, Proteintech Cat# 151121AP, Lot: 00073969), Anti-Psd95 (1:2000, Abcam Cat# ab18258, Lot: GR3258736-3), Anti-G3bp2 (1:2000, Abcam Cat# ab86135, Lot: GR3376678-6), Anti-b-actin (1:1000, Cell Signaling Cat# 3700, Lot: 8, Clone: 8H10D10), Anti-Synaptophysin (1:20,000, Abcam #ab32127, Lot: GR3270277-1, Clone: YE269), Anti-HDAC2 (1:1000, Cell Signaling Cat #2540S, Lot: 1). Primary antibodies used in this study for IF and brain tissue staining are as follows: Anti-Map2 (1:2000, Abcam Cat# ab5392, Lot: GR3450786-1), Anti-GFP (1:2000, Abcam Cat# ab6556, Lot: GR3459456-1), Anti-Sv2a (1:200, Abcam Cat# ab32942, Lot: GR3250909-1), Anti-Psd95 (1:2000, Abcam Cat# ab18258, Lot: GR3258736-3). Primary antibodies used in this study for immunoprecipitation are as follows: Anti-Caprin1 Antibody (2 ug, rabbit, Proteintech Cat# 151121AP, Lot: 00073969), Anti-FLAG (2 ug, Sigma Cat# F7425, Lot: 0000131574); Anti-IgG (2 ug, Cell Signaling Cat#2729, Lot: 6). Secondary antibodies used for western blot are Anti-Rabbit IRDye 800CW (1:15,000, Li-COR Cat#926-32211, Lot: C60816-02) and Anti-Mouse IRDye 800CW (1:15,000, Li-COR Cat#926-32212, Lot: C80108-05). Secondary antibodies used for IF and brain tissue staining are as follows: Anti-Chicken Alexa Fluor 488 (1:2000, Thermo Fisher Scientific Cat#A11039, Lot: 1599396), Anti-Rabbit Alexa Fluor 647 (1:1000, Thermo Fisher Scientific Cat#A21245, Lot: 1837984) and Anti-Rabbit Alexa Fluor 546 (1:500, Thermo Fisher Scientific Cat#A11035, Lot: 1904467).

Validation

Anti-Caprin1 antibody was validated in previous publication: PMID: 31272829. Anti-Sv2a was validated in previous publication: PMID: 32341095. Anti-FLAG was validated in previous publication: PMID: 14507915. All other antibodies were validated by others and the validation statement was listed on the manufacture's website:
-Anti-PSD95. As stated on Abcam website: Suitable for: ICC/IF, WB, IHC-P
-Anti-G3bp2. As stated on Abcam website: Suitable for: ICC/IF, WB, IP
-Anti-b-actin. As stated on Cell Signaling website: Applications: WB, W-S, IHC-P, IF-IC, FC-FP
-Anti-Synaptophysin. As stated on Abcam website: Suitable for: WB, IHC-P, ICC/IF
-Anti-HDAC2. As stated on Cell Signaling website: Applications: WB, IF-IC
-Anti-Map2. As stated on Abcam website: Suitable for: ICC, IHC-P, WB
-Anti-GFP. As stated on Abcam website: Suitable for: IHC-P, Electron Microscopy, ICC, IP, Flow Cyt, IHC-Fr, WB
-Anti-IgG was used in our RIP-qPCR assays as negative control to ensure enrichment and as stated on Cell Signaling website: Applications: IP, ChIP

Eukaryotic cell lines

Policy information about cell lines and Sex and Gender in Research

Cell line source(s)	HEK-293T cell line, ATCC Cat# CRL-11268
Authentication	None of the cell lines used were authenticated.
Mycoplasma contamination	All cell lines tested negative for mycoplasma contamination.
Commonly misidentified lines (See ICLAC register)	No commonly misidentified cell lines were used in this study

Animals and other research organisms

Policy information about studies involving animals; ARRIVE guidelines recommended for reporting animal research, and Sex and Gender in Research

Laboratory animals	10-14 weeks male C57BL/6 were purchased from Animal Resources Centre, Australia. Animals were maintained on a 12 h light/dark time schedule at 18-24 degree C and a relative humidity between 30-70% .
Wild animals	The study did not involve wild animals.
Reporting on sex	Findings apply to only male mice.
Field-collected samples	The study did not involve samples collected from the field.

Ethics oversight	All testing took place during the light phase in red-light-illuminated testing rooms following protocols approved by the Institutional Animal Care and Use Committee of the University of Queensland.

Note that full information on the approval of the study protocol must also be provided in the manuscript.
